# Percutaneous thrombectomy in the management of early rethrombosis in venous thoracic outlet syndrome: two case reports

**DOI:** 10.1186/s42155-021-00250-6

**Published:** 2021-08-06

**Authors:** Ján Sýkora, Kamil Zeleňák, Martin Vorčák, Adam Krkoška, Štefánia Vetešková, Martina Sýkorová, Jozef Sivák, Ľuboš Hlinka, Igor Šinák

**Affiliations:** 1 Clinic of Radiology, Comenius University’s Jessenius Faculty of Medicine and University Hospital, Kollárova 2, 036 59 Martin, Slovakia; 2 Vaša ambulancia Ltd., Prieložtek 1, 036 01 Martin, Slovakia; 3 Middle-Slovak Institute of Cardiovascular Diseases, Cesta k nemocnici 1, 974 01 Banská Bystrica, Slovakia; 4 Department of Vascular Surgery, University Hospital, Kollárova 2, 036 59 Martin, Slovakia

**Keywords:** Venous thoracic outlet syndrome, Paget-Schroetter syndrome, Upper extremity deep venous thrombosis, Percutaneous mechanical thrombectomy, Aspiration thrombectomy

## Abstract

**Background:**

Venous thoracic outlet syndrome resulting in the upper limb deep venous thrombosis is known as Paget–Schroetter syndrome or effort thrombosis. A general treatment algorithm includes catheter-directed thrombolysis followed by surgical thoracic outlet decompression.

There are limited data regarding endovascular treatment of rethrombosis presenting early after the surgery.

**Case presentation:**

Two cases of early rethrombosis successfully treated with percutaneous mechanical thrombectomy by two different techniques are described. In both cases, rethrombosis was diagnosed soon after thrombolysis and first rib resection with scalenectomy. After 12 months, both patients remain symptom-free, with patent subclavian veins confirmed by duplex ultrasonography.

**Conclusion:**

Percutaneous mechanical thrombectomy devices may offer a safe treatment option for patients with recurrent thrombosis after thoracic outlet surgery, even when thrombolytic therapy is contraindicated.

## Background

Venous thoracic outlet syndrome with acute axillo-subclavian vein thrombosis, also known as Paget–Schroetter syndrome or effort thrombosis is a rare condition, which accounts for approximately 1–4% of all deep venous thromboses (Illig and Doyle 2010).

Repeated compression of the subclavian vein within the costoclavicular space may cause venous injury with resultant stenosis and thrombosis. It predominantly affects young individuals. Typical symptoms on presentation include an arm swelling with associated pain, cyanosis, and engorged chest wall veins. Postthrombotic syndrome, as a late complication of upper-limb deep venous thrombosis, can significantly impact patients’ quality of life, especially if it occurs in the dominant arm. It comprises chronic pain, paraesthesia, heaviness, and functional limitation (Elman and Kahn 2006).

Treatment with anticoagulation alone may lead to residual chronic disability in up to 70% of patients (Schneider et al. 2003). A common strategy is to perform the catheter directed thrombolysis to clear most of the fresh clot. Non-surgical management with anticoagulation alone after initial endovascular treatment carries a risk of recurrent thrombosis ranging between 20 and 30% (Kärkkäinen et al. 2016), what justifies more aggressive operative thoracic outlet decompression.

We describe the use of percutaneous aspiration and mechanical thrombectomy in the successful management of two patients with recurrent thrombosis of the subclavian vein soon after thoracic outlet surgery.

## Case 1

A 21-year-old male, with no known previous medical illness, presented with a 9-days history of the non-dominant left arm swelling. Duplex ultrasonography examination revealed thrombosis of the left basilic vein from the upper arm level extending proximally to the left axillary and subclavian veins.

Catheter-directed thrombolysis was performed with recombinant tissue plasminogen activator (rtPA) in a dose of 1 mg/h. Recanalization of left basilic, axillary and subclavian veins was achieved after 48 h of thrombolysis with short residual stenosis located in the proximal subclavian vein. Balloon angioplasty was not performed prior the first rib resection.

**Fig. 1:**
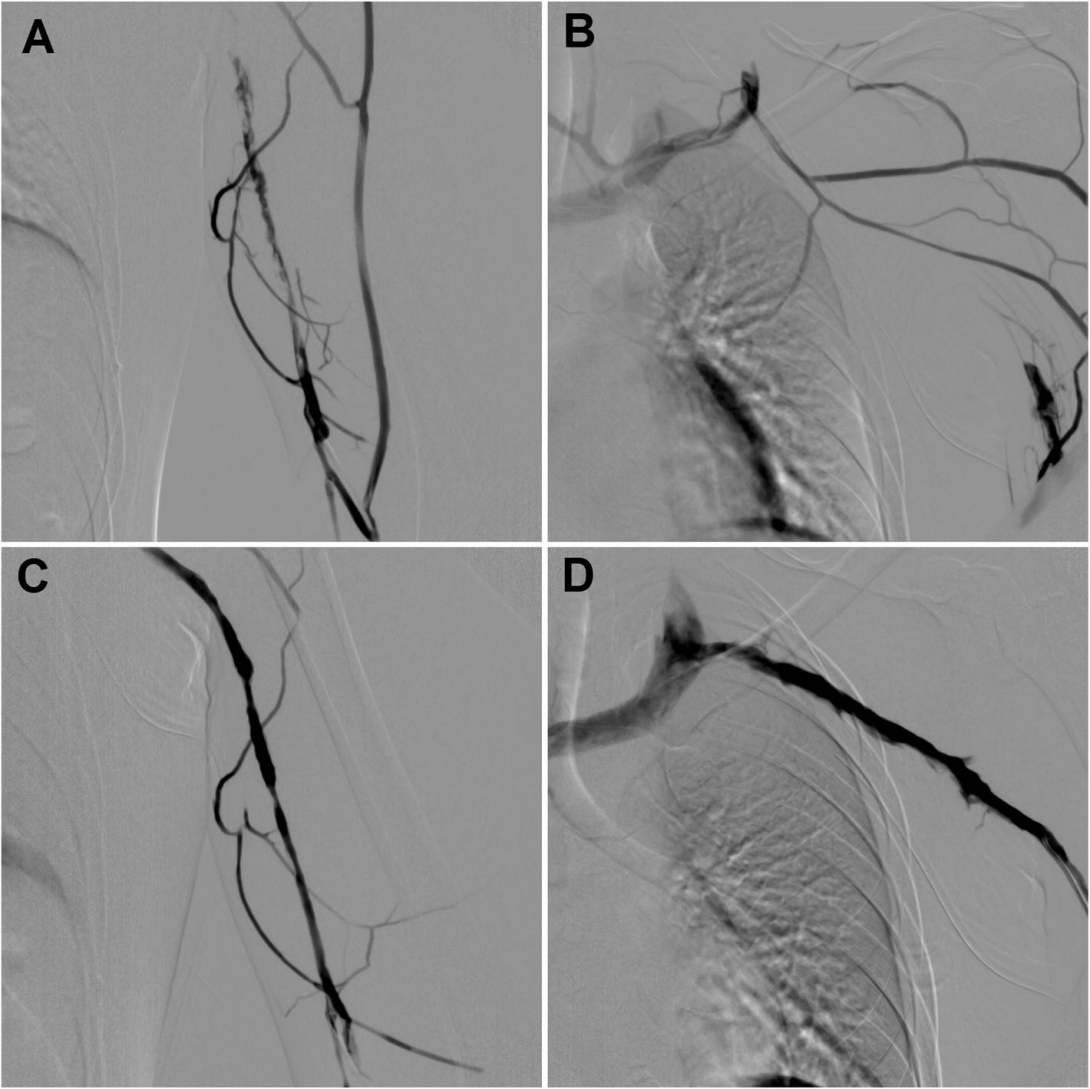
**A**, **B** Angiograms of a 21-year-old male with early rethrombosis of left basilic, axillary, and subclavian veins. **C**, **D** The final angiograms after percutaneous mechanical thrombectomy with Aspirex thrombectomy device confirmed recanalization of venous system

Significant swelling reduction of the arm was achieved after the thrombolysis. The patient was anticoagulated with continuous infusion of unfractionated heparin during the thrombolysis. The anticoagulation was switched to low-molecular-weight heparin (LMWH) afterwards. Thoracic outlet decompression surgery from transaxillary approach was performed the next day. Despite continued anticoagulation with therapeutic dose of LMWH after the 1st rib resection, routine angiography control 1 day after the surgery revealed early rethrombosis of left basilic, axillary and subclavian veins (Fig. 1A, B). Fluoroscopy confirmed adequate extend of 1st rib resection.

The right basilic vein was accessed, and percutaneous mechanical thrombectomy was performed. Aspirex 6F and 8F thrombectomy devices (Straub Medical, Wangs, Switzerland) were used to fragment the thrombus and to aspirate it from the vessel. Completion angiography showed restoration of venous patency and near complete thrombus removal, with only minor residual thrombosis of the subclavian vein, without significant stenosis (Fig. 1C, D). Haematological examinations did not reveal any hypercoagulable state. The patient was kept on LMWH for 1 month, followed by dabigatran for another 2 months. Long-term antiplatelet therapy with acetylsalicylic acid (ASA) was recommended afterwards.

During follow-up, patient’s arm had returned to normal, and he has remained asymptomatic. Duplex ultrasound controls 6 and 12 months after the procedure confirmed patent venous system.

## Case 2

The second patient was a 17-year-old female with a six-days history of swelling, and discolouration of the dominant right arm. Duplex ultrasound confirmed thrombosis of the right axillary and subclavian veins. Venography control after 48 h treatments with catheter-directed thrombolysis (rtPA, 1 mg/h) confirmed recanalization of the veins. Percutaneous angioplasty was then performed on the tight stenosis of the subclavian vein as it crossed the first rib using a 12-mm balloon catheter. The patient received anticoagulation treatment with unfractionated heparin during the thrombolysis and with LMWH afterwards.

**Fig. 2:**
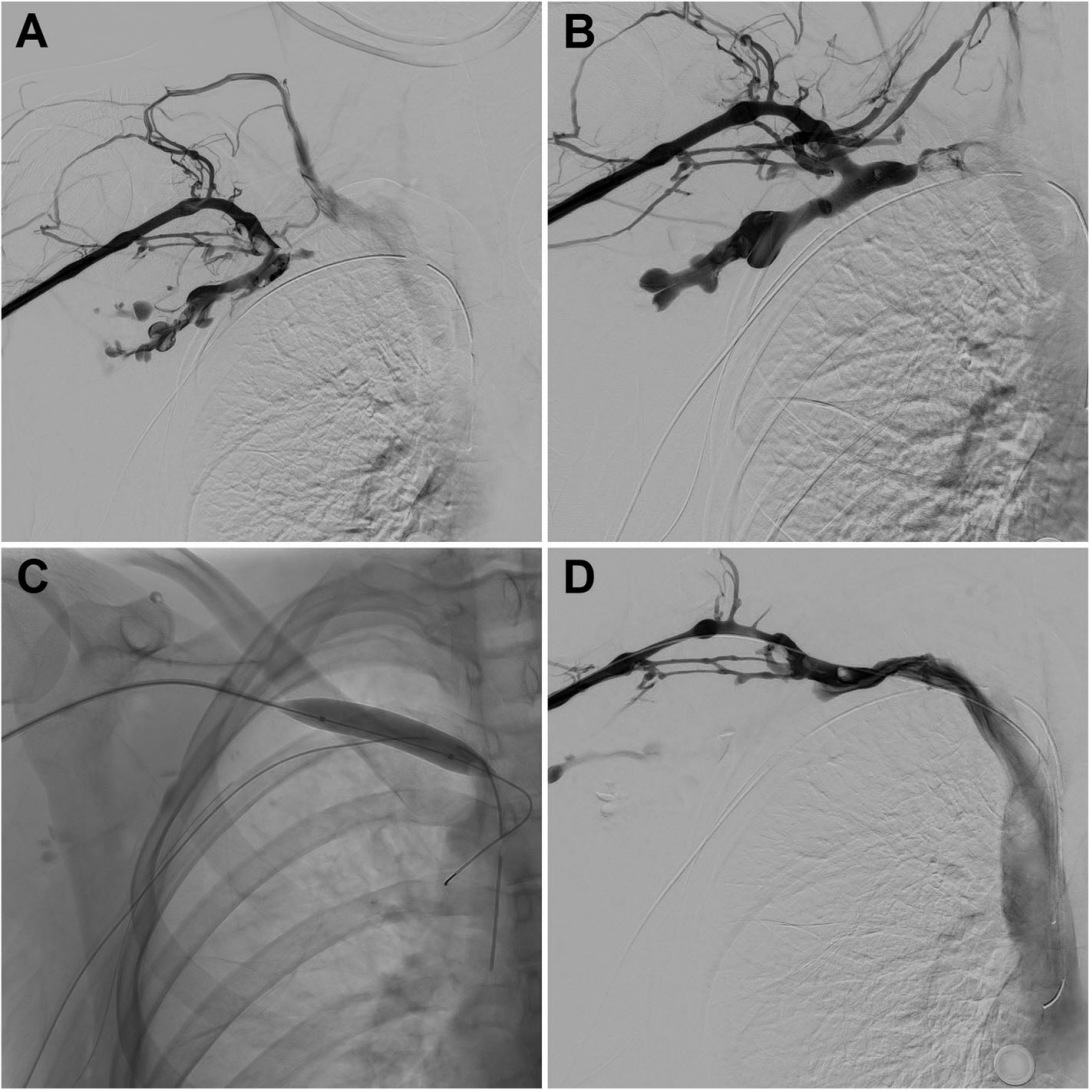
**A** Angiogram of a 17-years-old female with early recurrence of right subclavian vein thrombosis. **B** Recanalization of distal part of subclavian vein was achieved with Indigo Aspiration Catheter. **C** Angioplasty of the proximal subclavian vein. **D** The final angiogram after further aspiration confirmed recanalization of venous system

First rib resection and the scalenectomy was performed 3 days after the thrombolysis from transaxillary approach, and was confirmed by fluoroscopy. Repeating venography the following day demonstrated recurrent thrombosis of the right subclavian vein (Fig. 2A). The right cephalic vein was accessed, and Indigo Aspiration Catheters (Penumbra, Inc., Alameda, California, USA) and vacuum pump were used to aspirate the thrombus from the vessel. The Indigo Separator was used in tandem with the aspiration catheter to break up clot in the lumen of the catheter. Residual stenosis of the proximal subclavian vein was dilated with 10 mm angioplasty balloon to avoid excessive dilatation in the early post-operative period (Fig. 2C). Angiographic images post procedure showed good flow of contrast into the right subclavian and brachiocephalic veins (Fig. 2D). The use of stents even after surgical decompression remains controversial, and was not considered in our cases. According to the literature, stents may have a worse prognosis than balloon angioplasty alone (Illig and Doyle 2010).

After the procedure, the patient received anticoagulation treatment with LMWH for 1 month, followed by warfarin for another 2 months, and long-term antiplatelet therapy with ASA afterwards. Haematological examinations confirmed hypercoagulable state, sticky platelet syndrome type I. Patent right axillary and subclavian veins were confirmed by duplex ultrasonography 6 and 12 months later, and the patient remained symptom free.

## Discussion

Historically, effort thrombosis was treated with anticoagulation alone and the results have been disappointing. Catheter directed thrombolysis has shown nearly 100% successes in fresh clots if treatment was initiated within a few days of onset of symptoms, otherwise it has been successful in 62 to 84% of the cases (Doyle et al. 2007)*.*

Different pharmacomechanical thrombectomy devices have also been successfully used for the treatment of upper extremity deep venous thrombosis (Kärkkäinen et al. 2016; Mahmoud et al. 2018; Kim et al. 2006; Papantoniou et al. 2013). When there is a need to avoid or minimize exposure to rtPA, aspiration and mechanical thrombectomy systems can aspirate thrombus without the use of rtPA, resulting in decreased bleeding risk (Teter et al. 2020). Kosai described a case of Paget-Schroetter Syndrome treated with Aspirex thrombectomy device (Kosai et al. 2017)*.*

The feasibility and efficacy of aspiration thrombectomy prior the thoracic outlet surgery in Paget–Schroetter syndrome was recently demonstrated in Teter’s study. One-third of patients, primary treated with the Indigo continuous aspiration mechanical thrombectomy system, avoided any additional catheter-directed thrombolysis exposure. Only three out of sixteen patients required additional overnight thrombolytic therapy (Teter et al. 2020)*.*

There are limited data regarding endovascular treatment of early rethrombosis after surgical decompression in Paget-Schroetter Syndrome, when thrombolysis is contraindicated.

Shneider described a case of successful recanalization of rethrombosed subclavian vein 3 days after operative thoracic outlet decompression with AngioJet Rheolytic Thrombectomy System (Possis Medical Inc., Minneapolis, MN, USA) (Schneider et al. 2003).

## Conclusions

Two cases of early recurrent thrombosis successfully treated with percutaneous aspiration and mechanical thrombectomy soon after thoracic outlet surgery were described in our report. Both recanalization techniques were effective in thrombus removal. Percutaneous thrombectomy can avoid the hazards of open thrombectomy, when rethrombosis of the subclavian vein occurred in the postoperative period after thoracic outlet decompression procedures. These patients may be safely treated with percutaneous aspiration and mechanical thrombectomy techniques, even when thrombolytic therapy is contraindicated.

## Data Availability

The primary data underlying this manuscript are available from the corresponding author upon request.

## Abbreviations

rtPA: Recombinant tissue plasminogen activator
LMWH: Low-molecular-weight heparin
ASA: Acetylsalicylic acid
